# Application of Simulation Methods in Cervical Spine Dynamics

**DOI:** 10.1155/2020/7289648

**Published:** 2020-08-31

**Authors:** Meng-Si Sun, Xin-Yi Cai, Qing Liu, Cheng-Fei Du, Zhong-Jun Mo

**Affiliations:** ^1^Tianjin Key Laboratory for Advanced Mechatronic System Design and Intelligent Control, School of Mechanical Engineering, Tianjin University of Technology, Tianjin 300384, China; ^2^National Demonstration Center for Experimental Mechanical and Electrical Engineering Education, Tianjin University of Technology, Tianjin 300384, China; ^3^Beijing Key Laboratory of Rehabilitation Technical Aids for Old-Age Disability, Key Laboratory of Rehabilitation Technical Aids Technology and System of the Ministry of Civil Affairs, National Research Centre for Rehabilitation Technical Aids, Beijing 100176, China

## Abstract

Neck injury is one of the most frequent spine injuries due to the complex structure of the cervical spine. The high incidence of neck injuries in collision accidents can bring a heavy economic burden to the society. Therefore, knowing the potential mechanisms of cervical spine injury and dysfunction is significant for improving its prevention and treatment. The research on cervical spine dynamics mainly concerns the fields of automobile safety, aeronautics, and astronautics. Numerical simulation methods are beneficial to better understand the stresses and strains developed in soft tissues with investigators and have been roundly used in cervical biomechanics. In this article, the simulation methods for the development and application of cervical spine dynamic problems in the recent years have been reviewed. The study focused mainly on multibody and finite element models. The structure, material properties, and application fields, especially the whiplash injury, were analyzed in detail. It has been shown that simulation methods have made remarkable progress in the research of cervical dynamic injury mechanisms, and some suggestions on the research of cervical dynamics in the future have been proposed.

## 1. Introduction

The role of the human spine is to support the body and protect the spinal cord. Among all spinal injuries, cervical spine injuries are the most frequent and can be life-threatening [1, 2]. Von Koch et al. [3] thought that neck injuries occupy 50% among all traffic injuries with heavy economic burdens. In the UK, soft tissue neck injuries cost more than £1 billion a year to insurance companies, accounting for the most percent of the cost of personal injury claims [4]. Cervical spine biomechanics have been concentrated on both experimental and computational investigation with the aim to determine thphysical properties of its parts for better risk assessment and have a better understanding of the cause of whiplash injuries. Regarding issues related to cervical spine disorders, volunteer experiments are rare, costly, and limited, while on the other hand, cadaveric experiments are not representative in most cases. The multibody (MB) and finite element (FE) methods which were as computational techniques have been roundly adapted to develop the dynamics of the cervical spine [5]. Computational models exert a profound influence on a wide range of dynamic loading conditions.

Several review investigations that concern the computational development of the cervical spine have already been published [6–10]. The first review on material properties and validation data of experiments for cervical spine models was summarized by de Jager [6]. Huelke and Nusholtz [7] reviewed on the reasons and biomechanics of cervical spine impact injuries and tolerances of clinical and laboratory research reports. Among other biomechanical models, Panjabi [8] simply summarized FE models of the neck. Yoganandan et al. [9] concentrated on the progress in the development of models (geometry), constitutive law identification, and model calibration, which is considered as the most important phase. Fagan et al. [10] reviewed the development of finite element analysis for spinal modelling.

**Figure 1:**
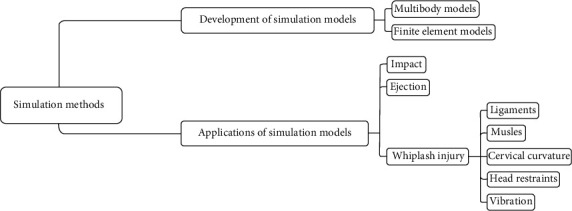
Main aspects of the review.

Although in the recent years, numerous reviews on several aspects related to the cervical spine have been published [6–12], a few recent literature reviews have focused on the application of simulation methods in cervical spine dynamics. Therefore, as shown in Figure 1, this paper reviewed the improvement of simulation models including new modeling information, such as the details of IVDs, ligaments, and muscles in the recent years, and focused on the application of simulation models under different dynamic conditions including impact, ejection, and whiplash injury caused by the impact. At last, further research on simulation models is also discussed.

## 2. Development of Simulation Models

In the recent years, simulation methods have been broadly used in research concerning cervical biomechanics in order to help researchers gain a deep insight into the potential mechanisms of cervical spine injury and dysfunction. The most common simulation methods employ either MB models or finite element models. In this section, the structure, material properties, and advantages and disadvantages of different models will be briefly reviewed.

### 2.1. Multibody Models

MB dynamics is one of the most effective methods to study the response of the cervical spine in an accelerated condition [13]. Multi-rigid-body models, which are based on the MB dynamics theory, can be constructed easily, calculated quickly, and simulated the kinematics and dynamics of head-neck precisely. For this reason, MB models have been widely used to simulate the entire cervical spine [14]. Previous MB models of the cervical spine are summarized in Table 1 [15–31].

Over the past years, several studies on frontal and lateral collisions have been conducted. Williams and Belytschko [15] developed the first complicated head-neck model to characterize the behavior of the living body with the implementation of active muscle. Deng and Goldsmith [17] proposed a 3D model of the human head, neck, and upper torso with muscles, for predicting its motion for any specified initial conditions. The abovementioned model could also be used for comparison with the results of other investigators. To assess the behavior of passengers in the neck area of a car crash, De Jager et al. [19] built a neck model that can be applied to software MADYMO. Nevertheless, it is still limited in the model validation for rear-end collisions. Another detailed MB head-and-neck model was developed by Van Lopik and Acar [27], where the connections of linear actuators characterized both active and passive behaviors, which allowed muscles to curve along the vertebrae when the neck is bent.

The model of De Jager et al. [19] has been modified by many researchers. For example, Yamazaki et al. [22] used data of volunteer experiment to improve Jager's model. More specifically, he modified the connection and bending properties of the whole vertebrae for increasing rigidity of the model and, then, used the model to investigate the influence of accelerations in different directions on the motion of cervical vertebrae. Moreover, the head and neck of Jager's global model were employed by Van den Kroonenberg et al. [21] in a MB rear-impact human-body model. The reactions of different severity conditions were compared approximately with volunteers and human cadavers. Nevertheless, at the time the study was conducted, the validation of that model was imperfect for lacking of available data of experiments. Van Der Horst [24] made modifications to Jager's detailed model, which was then implanted into the body model built in MADYMO. In that model, muscles were able to move along with the neck curvature, providing a more realistic muscle-force movement line.

In the rear-impact environment, head-neck models have made great improvements on the muscle and material. Jakobsson et al. [18] designed a MB model (C0-T1) to use in the sagittal plane; however, the time of the muscle reflexes was not taken into consideration [17]. Linder and Svensson [32] developed a mathematical model of neck rear impact that was used in the development of the BioRID dummy [33], an anthropometric test device. The neck was supplemented with two nonlinear stiffness muscle substitutes, which introduced a greater response than the available neck models at the time. Stemper et al. [26] defined both active and passive muscle properties in a head-neck model built in MADYMO. However, active muscle effects were not considered. Esat and Acar [34, 35] developed a novel MB model with extremely advanced material properties that can successfully reproduce the characteristic motion of the cervical spine with rear-end crash scenarios.

Considering the aeronautical field, Himmetoglu et al. [36] developed a biofidelic model with simple architecture, according to the anatomical parameters of the pilot in human dimensions of Chinese male pilot population, which could characterize the cooperation between the human body and the seat in rear collision for 50-percentile pilots. In the simulations, the established model was combined with a seat-helmet MB model and was validated through volunteer tests. It was demonstrated that the model could reveal the damage of head-neck during ejection.

### 2.2. Finite Element Models

Compared to MB models, FE models can offer more detailed information concerning local deformations and stress distribution; thus, stresses and strains in complicated structures, such as spinal bones and soft tissues, can be more veritably investigated. During the past 20 years, technology has progressed to such a point that more complicated three-dimensional finite element neck models with explicit geometries based on CT images have been developed and applied widely in the field of injury biomechanics, including impacts with different directions. CT plays an important role in the establishment of the finite element model. Not only the geometry but also the definition of the material properties could be derived from CT, in particular, for bone structures [37, 38]. In tomography, different tissues of the head and neck have different gray values because of their different densities. Mimics threshold segmentation is to separate and extract the corresponding tissues by using different gray values. To analyze the subtle biomechanical changes of the vertebral body, the CT value-based material property assignment method can be taken into consideration. Several scholars [39–41] reported that CT value-based material property assignment could offer more details than the traditional homogeneous assignment method. Those details could reflect the anisotropic biological characteristics of different bones. We import the model into the Mimics and choose the empirical formula we need, the materials of the vertebrae were assigned according to the formulas in Mimics, and the CT values were divided accordingly to distinguish the differences between different bones [42, 43]. The FE method has been widely adopted in the research of spine biomechanics, relating to clinical treatment [44–48], dynamic impact, and so on [13, 49]. A full summary of previous FE spine models can be found in Table 2.

The first FE model of the full cervical spine was modeled by Kleinberger [50]. This model incorporated the intervertebral disc and spinal ligaments, but lacked any representation of the musculature. In addition, materials of all were regarded as isotropic linear elastic, which is not sufficient for representing a majority of biological tissues. Dauvilliers et al. [51] developed a neck model taking into account the passive action of the muscle and confirming the stiffness features of the soft tissues. The vertebrae and head were regarded as rigid bodies, while the tissues between vertebrae were modeled with brick and spring elements. In 1998, Yang et al. [52] constructed a more complex anatomical head-neck FE model, where linear elastic-plastic materials for the vertebrae and linear viscoelastic materials for IVDs could be hit in the axis direction of front and postimpact.

Deng et al. [53] developed a FE model which was one of the most advanced cervical spine models at the time. More specifically, this was the first FE tissue model based on fundamental tissue properties rather than calibrated or assumed ones. It incorporated nonlinear viscoelastic material models to represent tissues under dynamic conditions. Another novelty of Deng's model was to include active muscle characteristics using the Hill muscle model, where the muscles were represented using two spring elements to allow for realistic muscle-force estimation when the neck was flexed. The model developed by Halldin et al. [64] was the first FE cervical spine model that detailed the upper cervical spine complex and incorporated some modelling techniques used in spinal segment modelling, such as the use of a composite annulus fibrosus. Active muscle response was added to this model by Brolin et al. [65].

In 2003, Yang and Yao [55] established the first cervical FE biomechanical model, which has been widely used in the automobile safety field in China, where the neck model was connected with a rigid dummy head (based on the Hybrid III 50th percentile dummy head form) to simulate and verify front collision. Two years later, Yang et al. [66] developed a head model which included skull and brain. The neck model established in 2003 was modified and coupled with the head model to simulate and verify front and rear impact. However, the geometric data of both models were purchased by the Viewpoint Company in the United States. At the same time, Eggers et al. [57] made some necessary changes to Yang's model [52], taking into account the compression and lateral bending of the cervical spine, and the ligament was considered as nonlinear. The lateral impact response of the head and neck, as well as the damage to the upper part of the cervical vertebra, was investigated. Then, the model was simulated to assess the response of soft tissue in frontal collisions.

**Figure 2:**
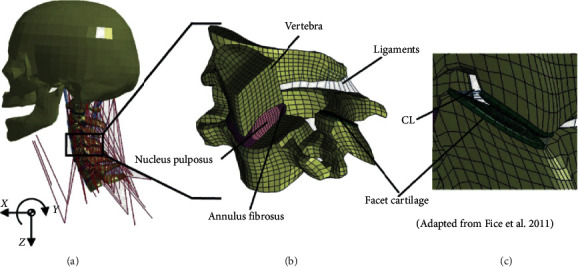
(a) Whole cervical spine model, (b) sectioned isometric view of the C4-C5 segment model, and (c) lateral close up of the C4-C5 facet joint. (Figure 2 is reproduced from Jason (B) [57] (under the creative commons attribution license/public domain)).

Meyer et al. [56] proposed a detailed FE neck model of a human volunteer and conducted an original model validation against experimental data recorded using the same volunteer. The temporal validation of the optimized model in the frequency domain demonstrated that adjusting the mechanical properties on the temporal response alone is not enough to ensure bio-faithful behaviour. The next year, Zhang et al. [1] developed a detailed C0-C7 FE model according to the precise geometries of a cadaveric specimen. A validation study was performed by simulating the model response under different typical loading conditions. Fice et al. [57] focused on a precise geometric and material representation at the tissue level to build a more representative FE model, which included more advanced ligament material properties [67]. As shown in Figure 2, the vertebrae geometries were in good agreement with the published anthropometry and were modeled as rigid bodies for computational efficiency. The intervertebral discs were constructed with solid elements for the annulus fibrosus ground substance and nucleus pulposus and layers of shell elements representing the fiber lamina (Figure 2(b)). The facet joints were represented with a superior and inferior layer of solid elements for the articular cartilage and a squeeze-film model to simulate the synovial fluid (Figure 2(c)). Ligaments were modeled using multiple 1D nonlinear rate-dependent tension-only spring elements. In addition, ligament relaxation was determined using an optimized method developed by Cronin et al. [60] to better predict neck kinematics and tissue level response.

Gender differences have been testified to be an significant cause for the occurrence of lasting whiplash-associated disorders (WAD). In particular, women are twice as likely as men on average to have sustaining symptoms and are more at risk under similar crash conditions [68, 69]. Consequently, Östh et al. [70] set up an average female FE model with ligaments for evaluating the rear-end collision biomechanics. Later, the model was combined with soft tissues to develop the head-and-neck model [61].

## 3. Applications

### 3.1. Impact

A number of studies on the response of the impact have been carried out, including dynamic responses of the neck, such as the neck load, angle of rotation, and acceleration, studied using multi-rigid-body models, as well as the analysis of local biomechanical responses and neck damage mechanisms, such as ligament elongation and intervertebral disc stresses, using finite element models based on human body anatomy (this part will be discussed in detail in Section 3.3).

The different responses to impact are affected by different impact accelerations. Zhang et al. [71] compared the predicted rotations, peak disc stresses, and ligament strains of each motion segment during whiplash, with and without acceleration exerted on the C7 inferior surface. Mustafy et al. [72] used a biofidelic model of the cervical functional spinal unit to compare the spinal load distribution under pure compression and sagittal flexion or extension at two different impact rates. Lee et al. [73] predicted changes in biomechanical parameters, including intradiscal pressure, dynamic stiffness, endplate region stresses, and the spine shock-absorbing mechanism under different impact durations or loading rates and investigated the relation between the loading rate and vertebral body fracture potential. White et al. [74] proposed an approach where the loading conditions from the simulations are fed into intervertebral disc FE models, which contribute to generating more realistic loading conditions. Hedenstierna et al. [75] investigated the distribution of neck muscle loads during impacts with different directions. The computed data, such as peak cross-sectional forces and effective strain distributions, were compared to experimental electromyography (EMG) data.

The models mentioned above indicated that the resistance of the whole spine to impact load depends on the loading rate and direction. In all spinal components where loads were applied, the load distribution of neck muscles was affected by load direction and the stress increased with the loading rate.

### 3.2. Ejection

Aircraft passengers who suffered with variable dynamic impacts are easy to develop cervical spondylosis or disabilities. Despite the fact that the ejection seat and restraint system have been developed over the past years, the occurrence of ejection related to spinal injury still maintains high. Many contributing factors add to the risk of injury during ejection, including the impact of axial (Gz) acceleration, the frequency of muscle endurance training, and the length of flight time [76]. Experimental work tends to be costly and well-prepared, while it just provides partial information concerning the reaction of the body under impact. Consequently, many research studies have focused on the mechanism of spinal cord injury emerged from impact loads or the use of numerical simulations to design protective devices [77].

The primary focus of the followings was to investigate the ejection trouble of the pilot. In 1958, Hess and Lombard [78] created the first continuum spinal model. In that model, the whole spine was regarded as a rod with homogeneous, linear elastic, free at the top, and a specified acceleration at the bottom. The most famous ejection model is the dynamic response index (DRI) model [6], which has been widely related to the data of injury and offers a helpful criterion for estimating safety under Gz acceleration. In ejection simulations, muscles need to be taken into account in order to better investigate the ejection mechanism. Soechting and Paslay [79] build a model to study the flexural response of the spine, which included spinal musculature effects. Since the global rigid dynamic models established in the previous studies could not provide the prediction of local stresses developed due to acceleration, Sadegh and Tchako [77] developed 3D FE models of the cervical spine capable of simulating the response of the neck muscular-skeletal structures when subjected to Gz acceleration forces during ejection.

Different ejection conditions may result in different cervical spine responses. Prasad and King [80] built a discrete human spine model to investigate the reaction of a pilot spine under a simulated vertical ejection. Teo et al. [81] used a detailed head-neck FE model, which had been previously validated, to study the effect of neck passive muscle strength on neck injury risk reduction during ejection. After the model was analyzed under a 10-G-ejection condition, it was further revealed that it is important for pilots to restrain muscle prior to ejection. According to the Life Mod, Song et al. [82] established a MB human-seat dynamic model to gain a deep insight into the mechanism of cervical injuries under various ejection environments.

Since both methods have different strong points and weak points in dealing with dynamic problems, their combination would contribute to investigate the spine in aviation conditions. Like the study of spine models for aviation safety by Kim [11], integrating the effect of the pilot belt, the FE model can provide more accurate analyses of the dynamic response of the spine after impact loading compared with the MB dynamics model.

### 3.3. Whiplash Injury

Whiplash and soft tissue sprains or strains of the cervical spine are the most frequent injuries in vehicle collisions, with nearly half of collision victims suffering this kind of injury [83, 84]. Despite the efforts of numerous researchers, the causative mechanism of whiplash injuries is not fully understood. In the numerical simulation field, the FE method offers an effective way to study human tolerance and potential injuries under collisions, especially in the condition of whiplash injury.

Several research groups have investigated whiplash using computational models of the cervical spine for automotive research [58, 85–88]. The review of the development of MB models and FE models has been summarized in Tables 1 and 2. In this section, the literature covering the use of numerical models to predict whiplash injury has been reviewed. The summary of these models reviewed is provided in Table 3, which gives a brief overview of each model, the validated loadings, injuries studied, and references.

#### 3.3.1. Ligaments

Ligaments are important soft tissues that have a certain effect on the whiplash injury. Since capsular ligament (CL) strain was recognized as one of the causes of prolonged pain for whiplash injury, Fice et al. [57] used the validated Panzer's model to simulate ligament strains for increasing rear impact severity. Their model was applied to research the distraction of ligaments and the probability of injury in the upper cervical spine. The simulation results showed that the upper cervical spine ligament strain increased with the increase of impact severity. The model proposed by Zhang was improved to incorporate the T1 vertebra [89] and, then, was used to investigate the cervical spine ligament tensions under different acceleration levels by applying different amplitude to the inferior T1 vertebral body. The results indicated that the peak impact acceleration plays an important role in the potential injury of the ligament. Cronin et al. [90] used a detailed numerical model to investigate the sources of pain generation under rear-end collisions. Ligament deformations are also considered as a mechanism causing whiplash injury. The model of human body (HUMOS) model developed by Tropiano et al. [87] was simulated to examine strain levels in ligaments and distinguish modes of injury. Obvious changes in soft tissue strains were observed at both the anterior and posterior cervical levels.

#### 3.3.2. Muscles

Whiplash simulations [87, 89, 90] involve not only the response of the ligamentous spine model but also the predicting of musculature with active or passive. Neck muscles also play a significant role in studying the whiplash injury. In 2002, Yoshida and Tsutsumi [91] analyzed the causes of such injuries using a FE model that incorporated muscle actions. The FE analysis results were compared to experimental results from volunteers [93] subjected to rear-end collisions, and it was revealed that the actions of the muscles affected the neck behavior of a driver involved in a rear-end car crash. Sharma et al. [94] used a newly developed active human skeletal muscle FE model to analyze the impacts of active muscle contraction on occupant kinematics under rear-end collision [95]. Simulations of 4 g rear impacts on the model exhibited that the muscle activation level can mimic the biofidelic behaviour of humans during crash. Similarly, Yan et al. [92] investigated the influence of active muscle force during neck injury. It was found that the active force emerged by the neck muscles in low-speed collision is more significant compared to that in high-speed collision. Recently, de Bruijn et al. [93] modeled a detailed finite element model with the muscle response validated to investigate head and neck motion during impacts. An OpenSim head-and-neck model was improved by Cazzola et al. [94] to investigate loading on the cervical spine during rugby, and then, Mortensen et al. [95] also modified OpenSim musculoskeletal models of the neck to study the moment generation and movement capabilities of Hyoid muscles.

#### 3.3.3. Cervical Curvature

Individual differences in cervical spine alignment of vehicle passengers are recognized to be a major factor for the high incidence of WAD in collision accidents. Studying the effect of spinal curvature on capsular ligament strains, the cervical lordosis curvature can be described as concave at the posterior surface, which is the normal curvature of a healthy individual. Stemper et al. [85] exposed their model to a simulated impact velocity of 2.6 m/s. The results demonstrated that, during a rear-impact, individuals with nonnormal spinal curvature were at a higher risk for sustaining CL injury, specifically in the lower cervical spine. In a separate study, Stemper et al. [86] employed the same model to predict anterior longitudinal ligament elongations during rear impact with up to 3.6 m/s velocity change and concluded that whiplash injury mitigation can be achieved by minimizing head retraction early in the impact. Similar to the latter study, a female full cervical spine model was used to study the impact of diverse spinal curvatures of the same individual on the motions and mechanical parameters relevant to WAD in rear impacts. Recently, Sato et al. [96] indicated that both volunteers including male and female exhibited diverse spinal alignment when they were in the same seat. Based on the results of the research by Östh et al. [61], we can predict that the geometry of automotive seats contributes to females gaining a cervical spine alignment with more ligament strain related to the neutral posture, which could be an explanation that females have a higher WAD risk than males. Individual differences can be represented through the differences of cervical spine alignment. The head-neck finite element model was, then, performed according to images of typical cervical spine alignments from a hospital database to obtain multiple head-neck finite element models with five different cervical spine alignments [97]. Rear-end impact analysis results showed that differences in the intervertebral rotation and ligament strain might be the cause of variability in the neck injury risk in rear-end impact accidents.

#### 3.3.4. Head Restraints

The occupant's response is influenced by a lot of factors including impact severity and seat design, as well as gender and posture. The majority of current finite element models are concentrated on modeling the head and neck, neglecting the interaction of the seat with the occupant during rear collision. In the 1960s, the head restraint was proposed as a measure in order to deal with the whiplash injury. For investigating the influence of head restraint backset on cervical spine kinematics in whiplash, a parametric study for improving the head restraint backset through exercising the model in rear impact was conducted by Stemper et al. [98] with a MADYMO head-neck model. Kitagawa et al. [58] used the THUMBS (Total Human Model for Safety human body model) to investigate the impact of an active head restraint on CL strain and neck injury criteria (NIC). It was found that, during rear impact, the peak capsular strain was significantly reduced with the use of an active head restraint. It was concluded by both Stemper and Kitagawa that the active head restraint was effective in reducing the risk of whiplash injury. In 2005, Hassan et al. [62] developed a comprehensive head-neck FE model to present the whiplash phenomenon in a rear-end collision environment, and the influence of the headrest on head-neck complex responses was also considered. The results showed that proper headrest can effectively keep away from extension injury during the acceleration stage of cervical spine in whiplash. Later, the Global Human Body Model Consortium (GHBMC) finite element model was applied to investigate these interaction impacts with emphases on the effect of seat belt, headrest, and seat stiffness on the occupant's response during rear-end collisions [99]. The study specifically indicates seat belts and headrests can effectively reduce damage caused by rear collisions and reduce head displacement and rotation.

### 3.4. Vibration

Long-term exposure to vibration may cause irreparable physiological or perception damages. According to the scientific literature [100], FE models have been used in vibration investigations; however, there are limitations, since the modelling is often restricted to the two-dimensional behaviour in the sagittal plane or spine models are often limited to the lumbar vertebrae and lack the cervical ones. The main problem for simulating the human vibration behavior is to consider the motion structure of bones and joints combined with the flexibility of soft tissues. Both Kong and Goel [101] and Pennestrì et al. [102] talked about the ability of FE models to predict the human vibration behavior. Kong and Goel [101] compared a model of the whole spine (head-sacrum) and a partial model (thorax-sacrum) and explained slight differences. Pennestrì et al. [102] performed a comparison between the FE model and a simplified MB dynamics model. They concluded that MB models can simulate underlying behaviors of vibration with an effective complexity. In 2003, Gonçalves and Ambrósio [103] proposed the combination of a MB model with a transport in order to evaluate the comfort of passengers. Then, Valentini and Vita [104] proposed a more explicit 3D MB model applied for the combination proposed by Gonçalves and Ambrósio [103]. In the vibration environment, muscles should be also taken into consideration. Therefore, Bazrgari et al. [105] evaluated the effect of muscle forces on systemic vibration behavior. It was summarized that the muscle counter reaction activity is very important in the high vibration amplitude environment. The models mentioned above with the description of the whole spine can present dynamics of the cervical spine in an ideal methods, including local responses, as well as specific contribution [101–105].

## 4. Conclusions

Numerical simulations have become an important research method for investigating human injury biomechanics. This paper reviewed the development of different human cervical spine computational models, mainly, MB and FE models, including the establishment of model geometry and the validation process, especially the application of simulation models. The literature involved in this paper provides deep insights into the understanding of the cervical spine injury mechanisms and related treatment and prevention. Despite the fact that the study of the cervical spine using simulation methods is continuously developing, there are still things that are imperfect. Therefore, some suggestions for further investigations in model construct are given as follows:

  In soft tissue modeling, most cervical spine models use 2D elements that the accurate anatomical features cannot be represented well. Developing more detailed and accurate 3D nonlinear models of soft tissues may contribute to truly predict of cervical spine responses.
  At present, the research in the field of vibration has been mainly focused on the lumbar vertebrae and not on the cervical spine. More efforts should be made to study, in depth, the mechanisms of damage based on cervical spine dynamics.
  In future studies, new material properties should be considered in order to obtain accurate dynamic responses of the cervical spine. Schroeder et al. [106, 107] developed the “OVED model” (osmo-poro-visco-hyper-elastic disc), which has lay a solid foundation for the precise determination of the biomechanical environments in the IVD.

The implementation of these recommendations for the development of future models can help us to construct a more perfect finite element model of cervical vertebrae, thus improving the accuracy and computational efficiency of the model. We can have a deeper understanding of the mechanism of cervical spine injury.

## Figures and Tables

**Table 1:** Overview of head-neck MB models.

Year	Author	Type	Segment	IVD details	Ligament details	Muscle details	Simulated impact cases
1983	Williams and Belytschko [15]	MB	C1-T1	6 DOF spring	Nonlinear springs	22 pairs, active (stretch-reflex)	Frontal-lateral
1984	Merrill et al. [16]	MB	C0-T1	6 DOF spring/damper	—	7 pairs, passive	Lateral-rear
1987	Deng and Goldsmith et al. [17]	MB	C1–C7	6 DOF spring/damper	—	13 pairs (3 Pt), passive	Lateral-rear
1994	Jakobsson et al. [18]	MB	Human body	—	—	Passive	Rear-end
1996	De Jager et al. [19]	MB	C0-T1	6 DOF spring/damper	Nonlinear viscoelastic	15 pairs, passive	Frontal-lateral
1997	Camacho et al. [20]	MB	C0-T1	3 DOF spring/damper	—	—	Axial
1998	Van den Kroonenberg et al. [21]	MB	Human body	—	—	Passive	Rear
2000	Yamazaki et al. [22]	MB	C0-T1	6 DOF spring/damper	Spring element	15 pairs, active (Hill)	Frontal-lateral
2000	Linder et al. [23]	MB	C0-T1	—	—	Muscle substitutes	Rear
2002	Van Der Horst et al. [24]	MB	C0-T1	6 DOF spring/damper	Nonlinear cables	68 pairs (multi Pt), active (Hill)	Frontal-rear
2004	Lee et al. [25]	MB	C0-T1	6 DOF spring	Nonlinear Cables	22 pairs (multi Pt), active (Hill)	Frontal
2004	Stemper et al. [26]	MB	C0-T1	—	Nonlinear Cables	Passive	Lateral-rear
2004	van Lopik et al. [27]	MB	C0-T1	—	Nonlinear viscoelastic	Passive, active (Hill)	Frontal-rear
2009	Himmetoglu et al. [28]	MB	Human body	Massless spring/damper	Massless spring-damper	Massless spring-damper	Rear
2016	Bruijn et al. [93]	MB	C0-T1	Spring/damper	Nonlinear Cables	Passive	—
2017	Cazzola et al. [94]	MB	Human body	—	—	MASI	Front
2018	Mortensen et al. [95]	MB	C0-T1	—	—	MASI	Lateral

DOF = degrees of freedom; Hill = Hill muscle model; Pt = points; IVD = intervertebral disk; MASI = Musculoskeletal model for the Analysis of Spinal Injuries.

**Table 2:** Overview of FE head-neck models.

Year	Author	Type	Segment	IVD details	Ligament details	Muscle details	Simulated impact cases
1993	Kleinberger [50]	FE	C0-T1	Isolinear IVD	Isolinear solid element	—	Frontal-axial
1994	Dauvilliers et al. [51]	FE	C0-T1	Linear composite	Linear spring/dampers	Passive	Frontal-lateral
1998	Yang et al. [52]	FE	C0-T1	Isolinear AF, viscoelastic NP	Linear cables and membranes	Passive	Axial-lateral
1999	Deng et al. [53]	FE	C0-T1	Isolinear AF, viscoelastic NP	Linear viscoelastic membranes	15 pairs, active (Hill)	Frontal
2000	Halldin and Brolin [54]	FE	C0-T1	Linear comp AF (shell)	Bilinear cables	14 pairs, active (Hill)	Frontal-lateral-axial
2003	Yang and Yao [55]	FE	C1–C7	Isolinear AF, viscoelastic NP	Spring elements	Hughes-Liu element, active	Frontal
2004	Meyer et al. [56]	FE	C0-T1	Isolinear IVD (AF and NP)	Nonlinear cables	Solid elements, passive	Frontal-lateral-rear
2005	Fice et al. [57]	FE	C0-T1	Isolinear AF, viscoelastic NP	Nonlinear tension-only membrane	—	Lateral
2006	Zhang et al. [1]	FE	C0–C7	Isolinear AF, Isolinear NP	Nonlinear cables	—	Rear
2008	Toyota Motor Corporation [58]	FE	Human body	—	Nonlinear tension-only membrane	Passive	Rear
2011	Panzer et al. [59]	FE	C0–C7	Solid hexahedral elements	Tension-only beam elements	25 pairs, passive, active (Hill)	Frontal
2011	Fice et al. [57]	FE	C0–C7	Solid elements	Nonlinear tension-only spring elements	25 pairs, passive, active (Hill)	Rear
2014	Cronin et al. [60]	FE	C0–C7	Isolinear AF, Isolinear NP	Nonlinear axial elements	Passive-active (Hill)	Rear
2016	Östh et al. [61]	FE	C0–C7	Hexahedral elements and orthotropic quadrilateral	Orthotropic membrane elements	Hill muscle	Rear
2017	Hassan et al. [62]	FE	Human body	Shell and brick elements	1D elements	1-D and brick elements	Rear
2018	Jiayi et al. [63]	FE	C0–C7	Isotropic linear elastic	Isotropic linear elastic (incompressible)	Passive (the Ogden model of superelastic materials)	Arrested landing

AF = annulus fibrosus; NP = nucleus pulposus; DOF = degrees of freedom; Hill = Hill muscle model; Pt = points; IVD = intervertebral disk.

**Table 3:** Overview of numerical models to predict whiplash injury.

Model name	Type	Description	Validated for	Injuries studied	References
TNO neck	MB	Skull to T1	Quasistatic facet response	ALL strain effect of posture of CL strain	[26, 85, 86]
		Vertebrae: rigid, scanned from cadaver	All response		
		Ligaments: piecewise linear springs	Rear impact		
		Discs: 3D point restraint			
		Facets: 1D point restraint			
		Muscle: 68 passive pairs			

HUMOS	FE	Full body 50th percentile male in the seated position	Quasistatic segment response	Vertebral stresses	[87]
		Approx. 50,000 elements	Frontal, oblique, and lateral impact	Ligament strain	
		Vertebrae: solid elastoplastic			
		Ligaments: 1D nonlinear springs			
		Discs: solid elements, incompressible fluid for nucleus, linear elastic for annulus			
		Facets: two layers of solid elements with springs for CL			
		Muscles: passive, nonlinear springs for elastic properties			

THUMS	FE	Full body 50th percentile male in the seated position	Quasistatic facet response	CL strains and NIC influence of active head restraints	[58]
		Approx. 80,000 elements	Rear impact		
		Vertebrae: linear elastic solids			
		Ligaments: piecewise linear discrete			
		Facets: no cartilage, shell elements for CL			
		Discs: solid linear elastic			
		Muscles: passive, 1D discrete			

ETH neck	FE	Skull to T1	Rear impact	Dynamic pressure spikes put the DRG at risk	[88]
		Vertebrae: rigid			
		Ligaments, facets, and discs modeled			
		Muscles: 1D discrete, active Hill type			

## Abbreviations

MB: Multibody
FE: Finite element
CL: Capsular ligament
WAD: Whiplash-associated disorders
2D: Two-dimensional
3D: Three-dimensional
IVD: Intervertebral disk
DOF: Degrees of freedom
AF: Annulus fibrosus
NP: Nucleus pulposus
EMG: Electromyography
DRI: Dynamic response index model.

## Additional Points

*Highlights*. (1) Cervical vertebrae are the most vulnerable to trauma after impact. (2) Simulation methods are widely used in the study of cervical injury mechanisms. (3) Review on the development process of related simulation models. (4) Detailed study on the application of simulation models in cervical dynamics. (5) Development and further challenges of cervical dynamics research.
